# Does Land-Use Intensification Decrease Plant Phylogenetic Diversity in Local Grasslands?

**DOI:** 10.1371/journal.pone.0103252

**Published:** 2014-07-25

**Authors:** Eugen Egorov, Daniel Prati, Walter Durka, Stefan Michalski, Markus Fischer, Barbara Schmitt, Stefan Blaser, Martin Brändle

**Affiliations:** 1 Department of Ecology, Faculty of Biology, Philipps Universität, Marburg, Germany; 2 Institute of Plant Sciences, University of Bern, Bern, Switzerland; 3 Department Community Ecology, Helmholtz Centre for Environmental Research, Halle, Germany; University of Tartu, Estonia

## Abstract

Phylogenetic diversity (PD) has been successfully used as a complement to classical measures of biological diversity such as species richness or functional diversity. By considering the phylogenetic history of species, PD broadly summarizes the trait space within a community. This covers amongst others complex physiological or biochemical traits that are often not considered in estimates of functional diversity, but may be important for the understanding of community assembly and the relationship between diversity and ecosystem functions. In this study we analyzed the relationship between PD of plant communities and land-use intensification in 150 local grassland plots in three regions in Germany. Specifically we asked whether PD decreases with land-use intensification and if so, whether the relationship is robust across different regions. Overall, we found that species richness decreased along land-use gradients the results however differed for common and rare species assemblages. PD only weakly decreased with increasing land-use intensity. The strength of the relationship thereby varied among regions and PD metrics used. From our results we suggest that there is no general relationship between PD and land-use intensification probably due to lack of phylogenetic conservatism in land-use sensitive traits. Nevertheless, we suggest that depending on specific regional idiosyncrasies the consideration of PD as a complement to other measures of diversity can be useful.

## Introduction

Land-use change is one of the primary drivers of biodiversity loss [1], [2]. Despite a large amount of studies dealing with the effects of land use on biodiversity, there are still gaps in the understanding of land use – biodiversity relationships. For example, the negative effects of different land-use types on biodiversity can differ in strength or vary in their effects. In addition, regional idiosyncrasies might interact with land use and affect biodiversity responses to land-use intensification, thus impeding general predictions [3]. Recent studies have advocated the consideration of phylogenetic diversity (PD) in ecological analyzes [4]–[7]. In brief, PD is defined as the total amount of phylogenetic space covered by species in a community. It therefore encapsulates the entire trait space of a community [8] and thus, may serve as a complement to trait diversity if the traits cannot be measured or trait data are not available [4]. Moreover, PD is an important factor for ecosystem function itself. It has been shown that PD can explain more variance in productivity in grasslands than species richness or functional diversity [9]. Plant productivity increased with mycorrhizal PD, which may be caused by niche differentiation, as increasing number of mycorrhizal families provide different advantages to their host plants [10]. Higher Plant PD also increases diversity of higher trophic levels and affects several ecosystem functions and processes [11]–[13]. That is, higher plant PD reinforces the positive effects of plant species richness on higher trophic levels when species richness is held constant [13]. Finally it has been found that PD promotes ecosystem stability and resilience [12] as well as interacts with plant species richness and alters its effect on herbivory [11]. Despite a consensus that PD is an important factor in understanding biodiversity – ecosystem functions relationships [7] or community assembly rules [14], little effort has been done in analyzing the effects of land-use intensity on PD [15].

In Central Europe managed grasslands are one of the most abundant and species-rich ecosystems [16]. In Germany, about 12% of area is covered by grasslands [17]. Most of these grasslands were established during a long period of low-intensity land-use and a large number of species have adapted to those conditions causing high levels of biodiversity. Land-use intensification in particular during the 20^th^ century posed considerable threats to biodiversity in grasslands, e.g. due to dramatic habitat loss and extinction of less competitive species [18]–[20]. It is also likely that land-use intensification will be the major driver of biodiversity loss in grasslands during the next decades [1], [21]. To attain a compromise between high land-use intensity and biodiversity conservation [1] and to assess the consequences of biodiversity loss a deeper understanding of the relationship between land-use intensification, biodiversity and ecosystem functioning is mandatory.

In general previous studies of plant biodiversity-ecosystem functioning relationships have shown that species richness enhances ecosystem functions [22]–[24]. Simply counting the number of species, however, is often not sufficient for analyzing the effects of biodiversity on ecosystem functions [25]. More comprehensive approaches consider functional diversity, defined as diversity of traits important for ecosystem level processes [26]. Functional diversity is thought to be the component of biodiversity with the largest effect on ecosystem processes [27]–[29]. However, implementation of trait data is subject to several limitations. For example, assessment of trait data is time-consuming and the *a priori* choice of specific traits is not always straightforward [26]. To overcome these shortcomings, PD has been proposed as a proxy for functional diversity [5], [30]. Recent studies, however, question PD as a proxy and propose it rather as a complement to functional diversity [31]. Despite the current discussion on the use of community phylogenetics in analyzes of assembly processes under several biotic and abiotic conditions [32] the importance of PD to ecosystem processes calls for its implementation into ecological analyzes [4]. While the negative effect of land-use intensification on species richness and functional diversity has been subject to many studies [2], [33], [34], a relatively small number of studies investigated how increasing land-use intensity affects PD of plant communities, particularly in grasslands. Studies that compared observed phylogenetic community structure of plants with expected patterns [30] revealed shifts in phylogenetic community structure with increasing disturbance and stress [15], [35]–[38]. Similar patterns were also shown within animal communities [39]–[41]. Changes in phylogenetic community structure may include shifts from overdispersion, where co-occurring species are less phylogenetically related than expected by chance, to clustering, where co-occurring species are phylogenetically more related than expected by chance. Such a shift from overdispersion to clustering is thought to be caused by environmental filtering that selects species with similar ecological traits that are likely to be closely related [15,36; but see 32]. Increasing land use intensity should therefore favor plant species with traits adapted to cope with effects of land-use intensification like fertilization, cattle grazing and frequent mowing. If such traits are phylogenetically conserved and play a major role in the phylogenetic community assembly, communities are likely to become phylogenetically more clustered with increasing land-use intensity. If traits are convergent or show a low phylogenetic signal, plant communities should not exhibit phylogenetic clustering with increasing land-use intensities or even lead to an increase in PD [38].

For conservational purposes the response of rare species to land-use intensification is of great interest. Rare species are in general more vulnerable to land-use intensification than common species [18]–[20]. Assuming that common species might be better adapted to high land-use intensities, phylogenetic diversity of common species should be less sensitive to land-use intensification than that of rare species. However, to our knowledge there are no studies exploring the response of PD of rare and common species to land-use intensification separately.

Socher et al. [3] showed that strength and direction of the effects of land use on biodiversity can differ between regions. Regional idiosyncrasies can also alter the effect of land use on phylogenetic diversity due to different regional species pools, environmental and geographical variables. It is therefore necessary to compare the effects of land-use intensification on PD among regions. Other limitations of previous research on plant PD are that the majority of studies are either experimental or describe phylogenetic patterns along natural or environmental gradients and are restricted to certain, often narrow, taxonomic scales [23], [42]. Descriptive studies of PD – land-use intensity relationships in human-disturbed systems are still scarce. When analyzing plant PD with respect to man-made disturbance, studies often focus on urban regions [37] or do not encompass the most common agricultural land-use categories such as fertilization, mowing and grazing. Including most common land-use types in descriptive studies of PD – land use relationships in agricultural systems could give new insights on these relationships under “real world” conditions. Previous studies may also suffer from the lack of considering species abundance data. Presence/absence data are highly sensitive to the chance and possible temporary occurrence of a single individual in unusual or unsuitable habitat. Interspecific relationships and interactions between species and ecosystems are based on interactions between individuals, which are cumulative in their effects. Neglecting abundance data may impede to discover important ecological relationships [6].

In this study we use species abundance data to analyze the PD of plant communities in local grasslands (150 sites) across land-use intensification gradients in three regions in Germany. In particular we aimed to answer the following questions:

1. Are there regional differences in the response of phylogenetic diversity to land use?
2. Does land-use intensification decrease phylogenetic diversity of plant communities in grasslands?
3. Does phylogenetic diversity of common and rare species assemblages show different relationships with respect to land-use intensification?

For a better understanding and interpretation of the relationship between PD and land-use intensification, information on the phylogenetic signals in traits relevant for landuse are of interest (i.e. related to a certain ecosystem function or environmental gradient). Thus, we used a set of traits that are likely to be sensitive toland use and tested for phylogenetic signal in those traits.

## Materials and Methods

### Study area

Our study is part of the Biodiversity Exploratories project, a large German research project to investigate the relationships between land-use, biodiversity and ecosystem functioning (www.biodiversity-exploratories.de). The Biodiversity Exploratories represent three typical regions in Germany covering a south-west – north-east gradient and each region comprises grasslands and forests under a range of land-use types and intensities [43]. The exploratory Schwäbische Alb (hereafter named Alb) is situated in the SW Germany and is part of the UNESCO Biosphere Reserve Schwäbische Alb. The exploratory Hainich-Dün (hereafter named Hainich) is situated in western Thuringia, central Germany. The exploratory Schorfheide-Chorin (hereafter named Schorfheide) is situated in NE Germany and is part of the UNESCO Biosphere Reserve Schorfheide-Chorin. In each region 50 experimental grassland plots representing gradients from semi-natural to intensive land-use were established (overall 150 plots). For more details see [43].

Field work permits were issued by the responsible state environmental offices of Baden-Württemberg, Thüringen, and Brandenburg (according to § 72 BbgNatSchG). The study did not involve protected or endangered species.

### Land-use

Land-use information for each of the 150 grassland plots was obtained by yearly interviews with farmers and land-owners between 2006 and 2010. The acquired information included fertilization level (kg nitrogen ha^−1^ year^−1^), mowing frequency (number of cuts year^−1^) and grazing intensity (livestock units×days of grazing ha^−1^ year^−1^) [43]. The three land-use components were standardized by the respective mean intensity within each region to yield the fertilization, mowing and grazing intensity [44]. For each year the individual components were summed up to a combined quantitative land-use intensity index (LUI). The yearly LUI-values (2006–2010) were averaged for each plot and the obtained means were then used in all our analyses [44].

### Vegetation releves and phylogeny

Between 2009 and 2011 we recorded the vegetation on a 4×4 m plot in each of the 150 grasslands three times (2009, 2010 and 2011). For each plot, vascular species richness and their relative abundance in percent cover was estimated. The species were further grouped into common and rare species based on their abundance for each year and region separately, taking into account local (plot) abundance and distribution (number of plots occupied) of each species. Common species were defined as the top 10% in terms of total abundance across plots occupied by a species, while the bottom 90% of the species were defined as rare. Based on these data we calculated the species richness of all, common and rare species as the average richness per plot across the three years. Note that the analyses of plant species richness from our study sites have been already published elsewhere [3], [45]. We included these results here only for comparative purposes. Therefore our discussion focuses only on the effects of land-use on PD. A low number of gymnosperms and ferns with low site incidence were omitted from all analyzes.

Phylogenetic relatedness of species was obtained from a well resolved and dated phylogeny of the Central European flora [46]. In brief, this phylogeny was assembled by manually grafting subtrees on a backbone topology, dating of nodes based on fossil records using the bladj algorithm in PHYLOCOM [47] and calculating an ultrametric tree (for details see [46]). We pruned the overall phylogeny to match the species pool of each of the three regions. As a result we obtained three trees, one for each region, representing the phylogenetic relationships of the respective species pool.

According to the data sharing regulations of the Biodiversity Exploratory Project and in accordance with the rules of the German Science Foundation DFG, the data will be made publicly available no later than five years after collection.

### Traits and phylogenetic signal

We compiled functional trait data from different data bases. As traits related to productivity we included the maximal plant height (cm) and specific leaf area (SLA; in cm^2^/g). As traits related to reproduction we used data start of flowering (month of the year). Data on the SLA were taken from the LEDA trait data base [48], data on start of flowering and plant height were gathered from BiolFlor data base [49] and from floras [50], [51]. Means were calculated when entries differed among the sources, but generally the values were highly consistent across sources. We further compiled performance and persistence traits relevant for agricultural grasslands: (1) soil nutrient indicator value (N, [52], (2) mowing tolerance (M), (3) grazing tolerance (G) and (4) trampling tolerance (T, all according to [53] from [54] and Briemle pers. comm.). For all traits we hypothesized that different agricultural use, in particular fertilization, mowing and grazing selects for species with different traits values. All indicators have numeric values ranging from 1 (low) to 9 (high). Available trait data ranged from 77% (SLA, height and flowering onset) to 86% (G) of the species.

We tested for the strength and significance of phylogenetic signals in traits using Pagel’s λ and Blomberg’s K implemented in the phytools package [55] in R. We log transformed values for the maximum height to achieve normality. It has been proposed that Pagel’s λ is an overall more robust metric than e.g. Blomberg’s K [56], however, in general both metrics revealed similar results.

### Phylogenetic diversity

Phylogenetic diversity estimates of plots were calculated with the “picante” package in R [57]. We calculated for each year and region separately the mean pairwise distance (MPD) and mean nearest taxon distance (MNTD) [30] weighted by species abundance (estimated % cover) as well as using presence/absence data. Considering % cover as a surrogate for species abundance may only approximate the “true” species abundance distribution within a community. However because of the large number of plots in our study individual counts of species would be very time-consuming and are thus not feasible. Estimates of % cover are at least rough approaches to estimate abundance and we suggest that analyses based on such approaches are more meaningful than considering only presence/absence data, especially in the context of the relative contribution of abundant, subordinate and transient species [59]. We used a slightly modified calculation of MPD based on abundance data as proposed by Gerhold et al. [60] to reduce effects of species richness. Abundance weighted and presence/absence versions of indices showed moderate correlations (MPD: r = 0.41; MNTD: r = 0.58). However, results based on the two indices did not differ considerably and therefore we present here only the results of abundance weighted indices (see Appendix S5 and S6 for presence/absence PD results). MPD measures the mean phylogenetic distance between two taxa in a sample and MNTD the mean phylogenetic distance to the nearest taxon in a sample. Hence MPD summarizes all phylogenetic distances including those of very distantly related species (e.g. between species of different orders) while MNTD considers only those between the most closely related species (e.g. between species within a genus). Thus, a stronger relationship of MNTD with land-use intensity compared to MPD would indicate that land-use has a stronger effect on the terminal than on the basal phylogenetic composition of a community. Both metrics depended on species richness and we therefore calculated standardized effect sizes ((observed metric - expected metric)/standard deviation of expected metric). We used a null model that shuffles the tip labels of the phylogeny maintaining all other properties of the sample matrix (i.e. species richness in plots and species prevalence). This null model was chosen since it tests for the null hypothesis, that phylogeny is not an important factor for structuring plants communities. Note that effect sizes of both metrics were calculated for each year and region separately. For each plot we then calculated averages across the three years which were further used in all subsequent analyses (see above).

We used simple linear regressions and ANOVAs to analyze the relationships between plant PD and land-use intensification. We considered region (exploratory) as a factor to analyze whether PD differs among regions and whether the relationships between PD and LUI differ among regions (region×LUI interaction). To assess whether rare species assemblages respond more strongly to increasing land use than common species, we compared the slope of the regression lines with an ANCOVA by testing the significance of the LUI×“rarity” interaction. All statistical analyses were conducted in R [58].

## Results

A total of 282 vascular plant species were recorded in the three regions from 2009 to 2010 (Appendix S1). We found depending on the considered species pool and the specific traits analyzed varying levels for Pagel’s λ and Blomberg’s K (Appendix S2). Based on Blomberg’s K we found no strong phylogenetic conservatism in analyzed traits (Appendix S2). This suggests that PD cannot be seen as an overall proxy for functional diversity along land-use gradients.

Average total, rare and common species richness differed among regions (Appendix S3). Total and rare species richness decreased with increasing LUI with regional effects modulating the response of. In two regions (Alb, Hainich) total and rare species richness decreased with increasing LUI while in Schorfheide no effect was observed. The relationship between common species and LUI showed very contrasting patterns between regions but there was no overall decrease in species richness (Appendix S3).

**Figure 1:**
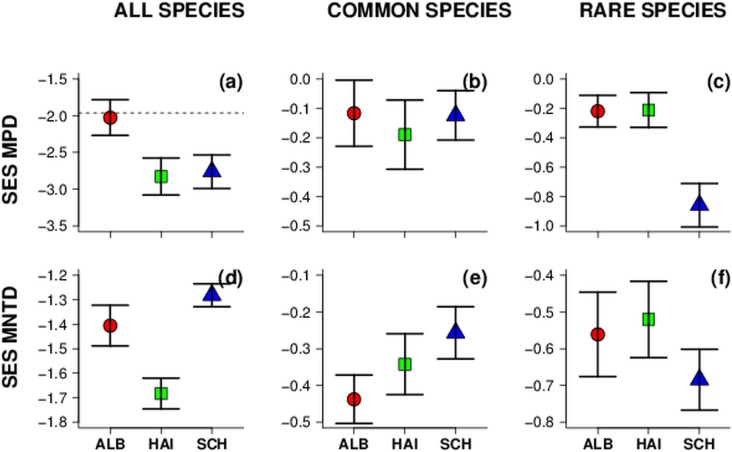
Mean (±SE) values of MPD and MNTD effect sizes for total, common and rare species assemblages in three regions in Germany. (a)–(c) Mean MPD and (d)–(f) mean MNTD for all, common and rare species assemblages in the three regions. Region abbreviations: ALB = SchwäbischeAlb (red circle); HAI = Hainich-Dün (green square); SCH = Schorfheide-Chorin (blue triangle). Error bars indicate ± SE. Points below the dashed line (<−1.96) are significantly clustered. Note different scales of y-axes.

**Figure 2:**
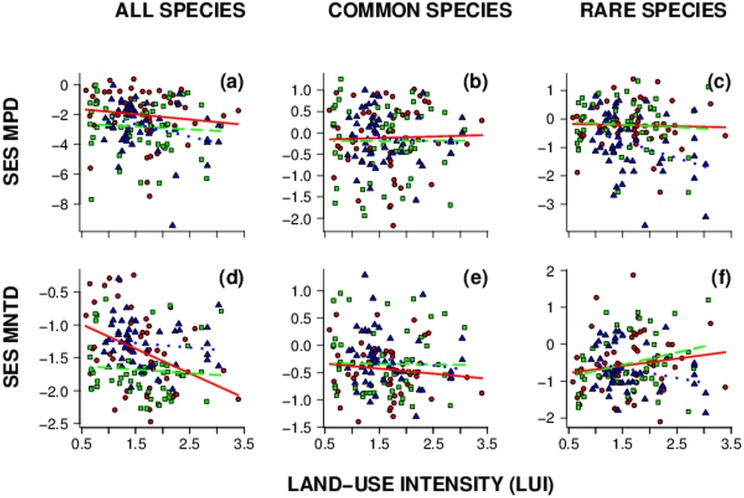
Relationships between mean pairwise distance (effect size MPD), mean nearest taxon distance (effect size MNTD) and land-use intensity (LUI) in three regions in Germany. Linear regression plots showing regression slopes for relationships between (a–c) mean pairwise distance and (d–f) mean nearest taxon distance for total, common and rare species assemblages and land-use intensity (LUI). Color and type code: red solid line/circle = Schwäbische Alb (Alb); green dashed line/square = Hainich-Dün (Hai); blue dotted line/triangle = Schorfheide-Chorin (Sch). Note different scales of y-axes. For significance of regression slopes see Appendix S4.

Overall, average PD strongly varied among regions. But note that the differences depended on the PD-metric used and whether rare/common species were considered (Fig. 1a–f). When all species were considered, effect size of MPD showed strong significant clustering of communities in two regions (Hainich and Schorfheide) while MNTD estimates showed random patterns in all three regions. Mean phylogenetic community structure was random in respect to phylogeny for common and rare species assemblages in all three regions. After accounting for regional differences, total species MNTD decreased with increasing land-use intensity while MPD showed only a marginally significant decrease with similar relationships in all three exploratories (Table 1). Furthermore, land-use had slightly different effects on MNTD depending on region indicated by a marginally significant region×LUI interaction (Table 1), with a stronger decline of MNTD in one region (Alb: r = −0.39, p<0.01, Appendix S4), in particular. The other two regions showed a non-significant negative trend (Fig. 2). For MPD, only one region (Schorfheide) showed a significant decline with increasing land-use intensity (r = −0.3, p<0.05; Fig. 1, Appendix S4).

**Table 1:** Effects of region, land-use intensity (LUI) and its interaction with region on effect size of (A) mean pairwise distance (MPD) and (B) mean nearest taxon distance (MNTD) for total, common and rare species assemblages.

A		MPD(total)		MPD(common)		MPD(rare)	
	df	F	p	F	p	F	p
Region	2	3.44	**0.035**	0.14	0.87	9.04	**0.0002**
LUI	1	3.65	0.06	0.01	0.91	3.71	0.06
Region×LUI	2	0.68	0.51	0.12	0.89	2.15	0.12
Residuals	144						
**B**		**MNTD(total)**		**MNTD(common)**		**MNTD(rare)**	
	**df**	**F**	**p**	**F**	**p**	**F**	**P**
Region	2	10.43	**<0.001**	1.51	0.22	0.73	0.48
LUI	1	7.60	**0.0066**	1.13	0.29	1.33	0.25
Region×LUI	2	3.02	0.052	0.25	0.78	3.51	**0.032**
Residuals	144						

ANOVA table with F and *p* values of the full models. Significant values in bold.

In general we found that for both common and rare species PD was not or only weakly affected by increasing land-use intensity. The relationships did not vary among regions except for rare species MPD (Table 1, Fig. 2). Overall, the strength of phylodiversity – land-use intensity relationships did not differ between common and rare species assemblages over three regions as indicated by non-significant LUI×rarity interaction terms in our models (Table 2).

**Table 2:** Values for t-statistics and corresponding p values of the linear models with (A) MPD and (B) MNTD as dependent variables and LUI, rarity (two-level factor: common and rare) and their interaction as independent variables.

A	MPD							
	ALL		ALB		HAI		SCH	
	t	p	t	p	t	p	t	p
Intercept	−0.65	0.52	−0.55	0.58	−0.61	0.54	0.04	0.97
LUI	−0.10	0.92	0.19	0.85	0.03	0.98	−0.42	0.67
Rarity	0.25	0.8	0.04	0.97	0.29	0.77	0.19	0.85
LUI×Rarity	−1.39	0.17	−0.30	0.77	−0.36	0.72	−1.80	0.08
**B**	**MNTD**							
	**ALL**		**ALB**		**HAI**		**SCH**	
	**t**	**p**	**t**	**p**	**t**	**p**	**t**	**p**
Intercept	−1.58	0.12	−1.09	0.28	−1.30	0.20	−0.21	0.83
LUI	−0.87	0.38	−0.61	0.55	−0.08	0.94	−0.98	0.33
Rarity	−2.61	**0.009**	−1.57	0.12	−2.08	**0.041**	−0.66	0.51
LUI×Rarity	1.52	0.13	1.33	0.19	1.69	0.09	−0.72	0.47

Interaction term determines whether rare species PD response differs from that of common species PD. Significant values in bold.

## Discussion

Land-use intensification is one of the major threats to global biodiversity in grasslands [21]. However, only a few studies have analyzed the effects of anthropogenic influence on PD of grassland plant communities. Several studies showed that anthropogenic influence can cause a decline in PD of species communities [15], [37], [61] which possibly may also decrease trait diversity and associated ecological functions [7]. In particular, PD can be important for ecosystem functioning when the ultimate processes, which depend on plant traits and trophic interactions, show a phylogenetic signal [7]. It has been shown that in grasslands PD can act as a better predictor of productivity than species richness or functional diversity [9], [62]. Moreover, herbivory was stronger related to phylogenetic relatedness than to plant functional traits [63]. An experimental study by Pellissier et al. [38] revealed an increase in PD after strong fertilization and herbicide application while functional traits showed contrasting relationships presumably by selecting for convergent traits [38]. We found no evidence for strong phylogenetic signal in selected land-use sensitive traits (Appendix S2). Thus, phylogenetic diversity may not capture the relevant functional information leading to a relatively weak response to land-use intensification [31]. On the other side, the significant decrease of PD depending on region and metric used (see below), shows that PD might capture additional information beside the measured traits.

Dinnage [15] showed that the phylogenetic structure of plant communities in disturbed plots of old field sites is more clustered than expected, whereas phylogenetic structure in undisturbed plots does not differ from random expectations. This indicates, that land-use might act similarly to environmental filters and select for (presumably closely related) species with similar traits, which enable species to cope with disturbance. However, Dinnage analyzed the vegetation of an old field system with plowing being the disturbance that affected the phylogenetic diversity. This kind of disturbance mediates phylogenetic succession which can lead to increasing phylogenetic clustering of plant communities [64]. Our study sites are exposed to land-use types completely different to the former study and our results differ in the strength of the PD response to land-use intensification. Although land-use intensification slightly decreased phylogenetic diversity, considering the mean nearest taxon distance (MNTD) in particular, it did not lead to a shift form random to clustered community structures (Table 1, Fig. 2). In general, plant communities exhibited clustered and random phylogenetic structures on plots with both, low as well as high land-use intensities (points <1.96 on y-axis; Fig. 2). There are factors causing clustering of communities, especially when considering the tree-wide patterns (MPD, Fig. 1a) as was shown in several studies [e.g. 5,35]. Whether these factors refer to environmental filters [65], [66] or exclusion of weak competitors [32] we cannot distinguish in our study. Land-use intensity, however, seems to play a minor role as determinant of phylogenetic community structure of plants in grasslands. This is contrary to the results of Dinnage [15] but such differences might be caused by different land-use types, with plowing causing a strong disturbance within habitats compared to our land-use types. Note also that in Dinnages study [11] no gradient of land-use intensity was analyzed and the definition of regional species pools was different from our study. Nevertheless, the slight decline of PD in our study may indicate that the influence of factors causing phylogenetic clustering of communities is mediated through or caused by increasing land-use intensity.

Many studies dealing with phylogenetic community structure use only one phylogenetic diversity index like NRI or NTI (equivalent to (−1 * effect size MPD) and (−1 * effect size MNTD), respectively) [e.g. 35,56]. Since the two metrics measure PD at different depths of phylogeny, with MPD (NRI) capturing tree-wide patterns and MNTD (NTI) being more sensitive to the tips of a phylogeny [30], depending on the distribution of traits, results of analyses might differ. However, when both metrics were used, similar results were reported [e.g. 57]. In our study, although the two metrics showed similar relationships with land use, MNTD was more sensitive to increasing land-use intensity. This emphasizes the importance of including different indices into analyzes of PD, as land-use sensitive traits might be conserved within a few relatively young clades (e.g. within families) and thus might be masked when using metrics considering a broader phylogenetic scale (e.g. MPD). Because MNTD shows a stronger response to land-use intensification it is possible that those traits are conserved in the younger nodes of phylogeny. Thus, using MPD might not capture relevant trait information when analyzing the effects of land use on phylogenetic diversity. In fact, as Blomberg’s K can be thought of as the partitioning of variance with low values (K<1) indicating variance within clades, this might be the reason for MNTD being more sensitive to land use.

Although common and rare species might differ in several traits [67] or their sensitivity to soil biogeochemical parameters [68] and respond differently to land use and competition [69], we found no significant differences in their response to increasing land-use using analysis of covariance (Fig. 2, Tab 2). This suggests that traits that probably affect the abundance of species are randomly distributed across our plant phylogeny or/and are not affected by land-use. The only trait that was relatively strong conserved in both, common and rare species, was maximum height. Despite a relatively high phylogenetic signal in this trait, it seems that height is not a strong determinant of phylogenetic community structure in both, common and rare species assemblages. Another explanation might be that PD of common and rare species might respond differently to the single LUI components due to different traits not accounted for in our study and combining those to one index might neglect the differences in strength and direction of responses. Likewise, as the effects of land-use on PD did not differ in general between common and rare species communities, but rather showed slightly different patterns on a smaller scale, they should be examined separately if conservation efforts attempt to increase diversity for endangered taxa.

It is well known that regional peculiarities and species pools influence regional phylogenetic diversity [70], [71]. For our study regions we found that considering all species Alb had overall high and Hainich overall low PD. Schorfheide showed contrasting patterns depending on the PD-metric used. Low MPD values suggest, that species in communities are closely related when accounting for the whole phylogeny, but high MNTD values indicate, that on lower phylogenetic scales (e.g. within families) species are distantly related. This might be explained by the fact that Schorfheide was more strongly affected by the Pleistocene glaciations than the other regions. One may argue that the plant communities of Schorfheide are still dominated by ecologically similar species belonging to closely related higher clades. Environmental filtering is then likely to cause strong phylogenetic clustering of communities considering the MPD (Fig. 1a). By contrast, within these clades PD might have increased due to limiting similarity [72] causing random community structure (Fig. 1d).

Differences in PD among regions may, to some extent, be also due historical land use rather than current [55] as suggested for species richness or functional diversity [70], [73]. Such regional differences call for a careful consideration of regional particularities when providing management strategies to maintain or increase phylogenetic diversity of grassland plant communities under “real world” conditions.

The theory behind phylogenetic patterns along disturbance gradients relies on several hypotheses about distribution of ecological traits across phylogenetic trees [9], [30], [32], [62], [74]. We showed that although potentially land-use relevant traits show some levels of phylogenetic conservatism, PD still can provide additional information. The consideration of PD is therefore in particular importantin situations when functional traits of species are not available. Phylogenetic methods can complement ecological analyzes, but it must be pointed out that PD cannot be seen as a surrogate for other biodiversity metrics, functional diversity in particular.

## Supporting Information

Appendix S1 Phylogenetic tree of plants in three regions in Germany used in this study.
(TIFF)

Appendix S2 Phylogenetic signal in 7 traits considered as sensitive to land use for all, common and rare species in the three regions (ALB: Schwäbische Alb, HAI: Hainich-Dün and SCH: Schorfheide-Chorin) and in all regions combined. Significant values are in bold.
(DOCX)

Appendix S3 Mean (±SE) values and regression slopes of species richness for total, common and rare species assemblages in three regions in Germany.
(TIF)

Appendix S4 Correlation coefficients and significance of the respective regression slopes from Fig. 1 and Appendix S3 and for all regions combined.
(DOCX)

Appendix S5 Relationships between presence/absence based mean pairwise distance (effect size MPD), mean nearest taxon distance (effect size MNTD) and land-use intensity (LUI) in three regions in Germany.
(TIF)

Appendix S6 Mean (±SE) values of presence/absence based MPD and MNTD effect sizes for total, common and rare species assemblages in three regions in Germany.
(TIF)
