# Supplementary material for: Does Land-Use Intensification Decrease Plant Phylogenetic Diversity in Local Grasslands?
Source: PLoS One. 2014 Jul 25;9(7):e103252. doi: 10.1371/journal.pone.0103252 (PMC4111588; doi:10.1371/journal.pone.0103252)
Supplement: Appendix S2 — Phylogenetic signal in 7 traits considered as sensitive to land use for all, common and rare species in the three regions (ALB: Schwäbische Alb, HAI: Hainich-Dün and SCH: Schorfheide-Chorin) and in all regions combined. Significant values are in bold. (DOCX) [file pone.0103252.s002.docx]

**Table S2. Phylogenetic signal in 7 traits considered as sensitive to land use for all, common and rare species in the three regions (ALB: Schwäbische Alb, HAI: Hainich-Dün and SCH: Schorfheide-Chorin) and in all regions combined. Significant values are in bold.**

|  |  | **All species** | |  | **Common species** | |  | **Rare species** | |
| --- | --- | --- | --- | --- | --- | --- | --- | --- | --- |
|  |  | **K** | **λ** |  | **K** | **λ** |  | **K** | **λ** |
| **SLA** | ALL | 0.099 | **0.297** |  | 0.138 | <0.001 |  | 0.097 | **0.182** |
|  | ALB | **0.165** | **0.208** |  | 0.172 | 0.081 |  | **0.16** | 0.175 |
|  | HAI | 0.075 | <0.001 |  | 0.143 | <0.001 |  | 0.074 | <0.001 |
|  | SCH | 0.1 | <0.001 |  | 0.12 | <0.001 |  | 0.115 | <0.001 |
| **log(max height)** | ALL | **0.167** | **0.636** |  | **0.267** | **0.735** |  | **0.297** | **0.93** |
|  | ALB | **0.158** | **0.712** |  | 0.309 | **0.855** |  | 0.135 | **0.646** |
|  | HAI | **0.188** | **0.665** |  | **0.42** | **0.726** |  | **0.172** | **0.603** |
|  | SCH | **0.238** | **0.614** |  | **0.886** | **0.805** |  | **0.238** | **0.531** |
| **Flowering onset** | ALL | **0.209** | **0.702** |  | 0.108 | <0.001 |  | **0.226** | **0.643** |
|  | ALB | **0.236** | **0.619** |  | 0.091 | <0.001 |  | **0.252** | **0.546** |
|  | HAI | **0.229** | **0.716** |  | 0.086 | <0.001 |  | **0.254** | **0.69** |
|  | SCH | **0.177** | **0.483** |  | 0.164 | <0.001 |  | **0.194** | **0.421** |
| **N** | ALL | 0.086 | **0.192** |  | 0.213 | 0.052 |  | **0.141** | 0.147 |
|  | ALB | 0.07 | 0.254 |  | 0.207 | 0.143 |  | 0.147 | 0.263 |
|  | HAI | 0.062 | <0.001 |  | 0.15 | <0.001 |  | 0.12 | 0.025 |
|  | SCH | 0.127 | <0.001 |  | 0.204 | <0.001 |  | 0.156 | <0.001 |
| **M** | ALL | 0.109 | **0.287** |  | 0.152 | <0.001 |  | **0.138** | **0.153** |
|  | ALB | 0.102 | **0.356** |  | 0.114 | <0.001 |  | **0.174** | **0.271** |
|  | HAI | 0.094 | **0.223** |  | 0.144 | <0.001 |  | 0.13 | 0.14 |
|  | SCH | **0.159** | **0.219** |  | 0.14 | <0.001 |  | 0.144 | 0.048 |
| **G** | ALL | 0.104 | **0.461** |  | 0.111 | <0.001 |  | **0.137** | **0.424** |
|  | ALB | **0.15** | **0.647** |  | 0.102 | <0.001 |  | **0.3** | **0.71** |
|  | HAI | 0.101 | **0.425** |  | 0.091 | <0.001 |  | 0.145 | **0.437** |
|  | SCH | 0.112 | 0.191 |  | 0.191 | <0.001 |  | 0.141 | **0.282** |
| **T** | ALL | **0.105** | **0.223** |  | 0.187 | <0.001 |  | 0.122 | **0.121** |
|  | ALB | 0.109 | **0.335** |  | 0.178 | <0.001 |  | 0.137 | 0.232 |
|  | HAI | 0.093 | **0.167** |  | 0.164 | <0.001 |  | 0.115 | 0.14 |
|  | SCH | **0.147** | **0.148** |  | 0.186 | <0.001 |  | **0.178** | 0.149 |
