# Supplementary figures and images for: Does Land-Use Intensification Decrease Plant Phylogenetic Diversity in Local Grasslands?
Source: PLoS One. 2014 Jul 25;9(7):e103252. doi: 10.1371/journal.pone.0103252 (PMC4111588; doi:10.1371/journal.pone.0103252)

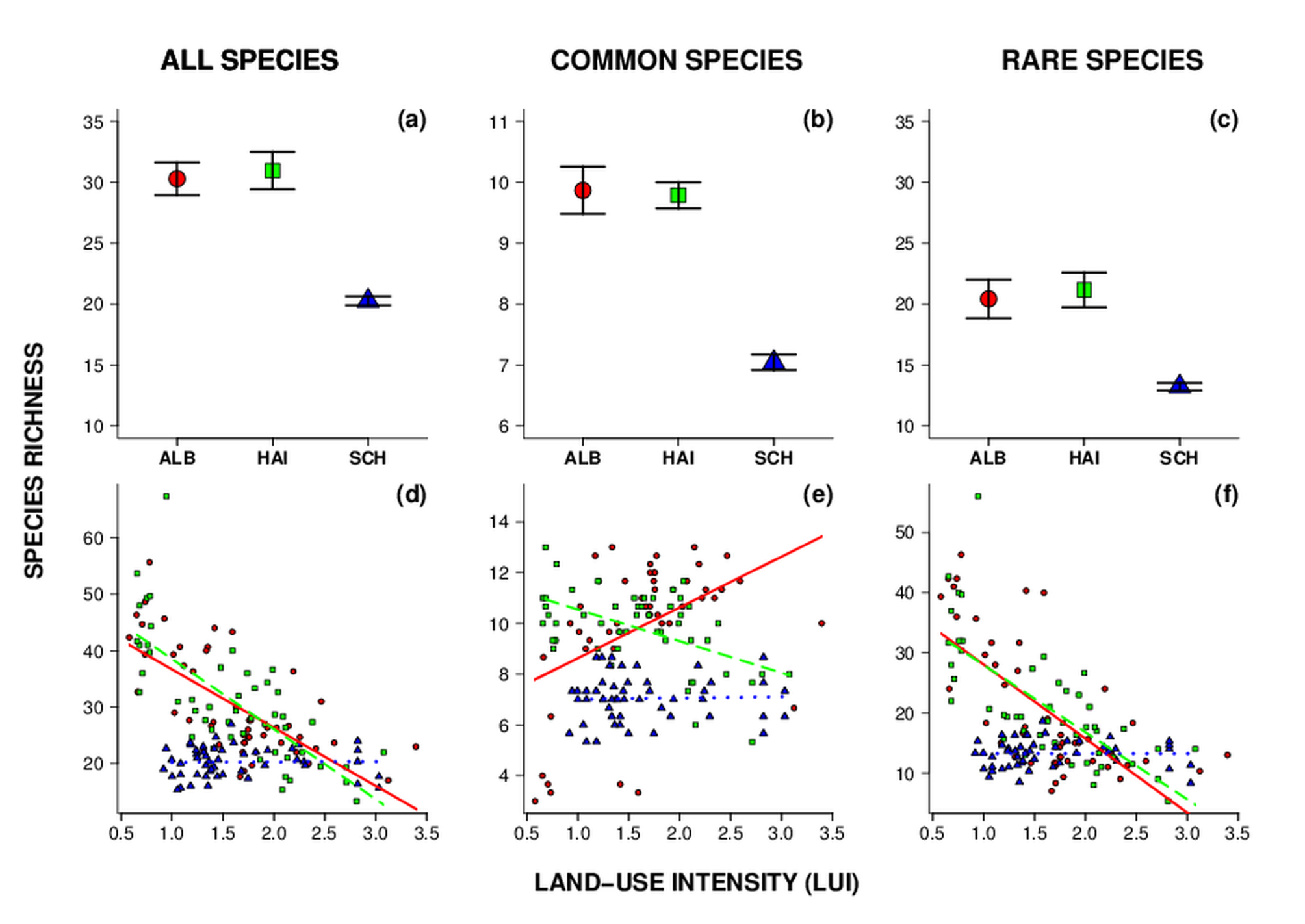

Supplement: Appendix S3 — Mean (±SE) values and regression slopes of species richness for total, common and rare species assemblages in three regions in Germany. (TIF) [file pone.0103252.s003.tif]

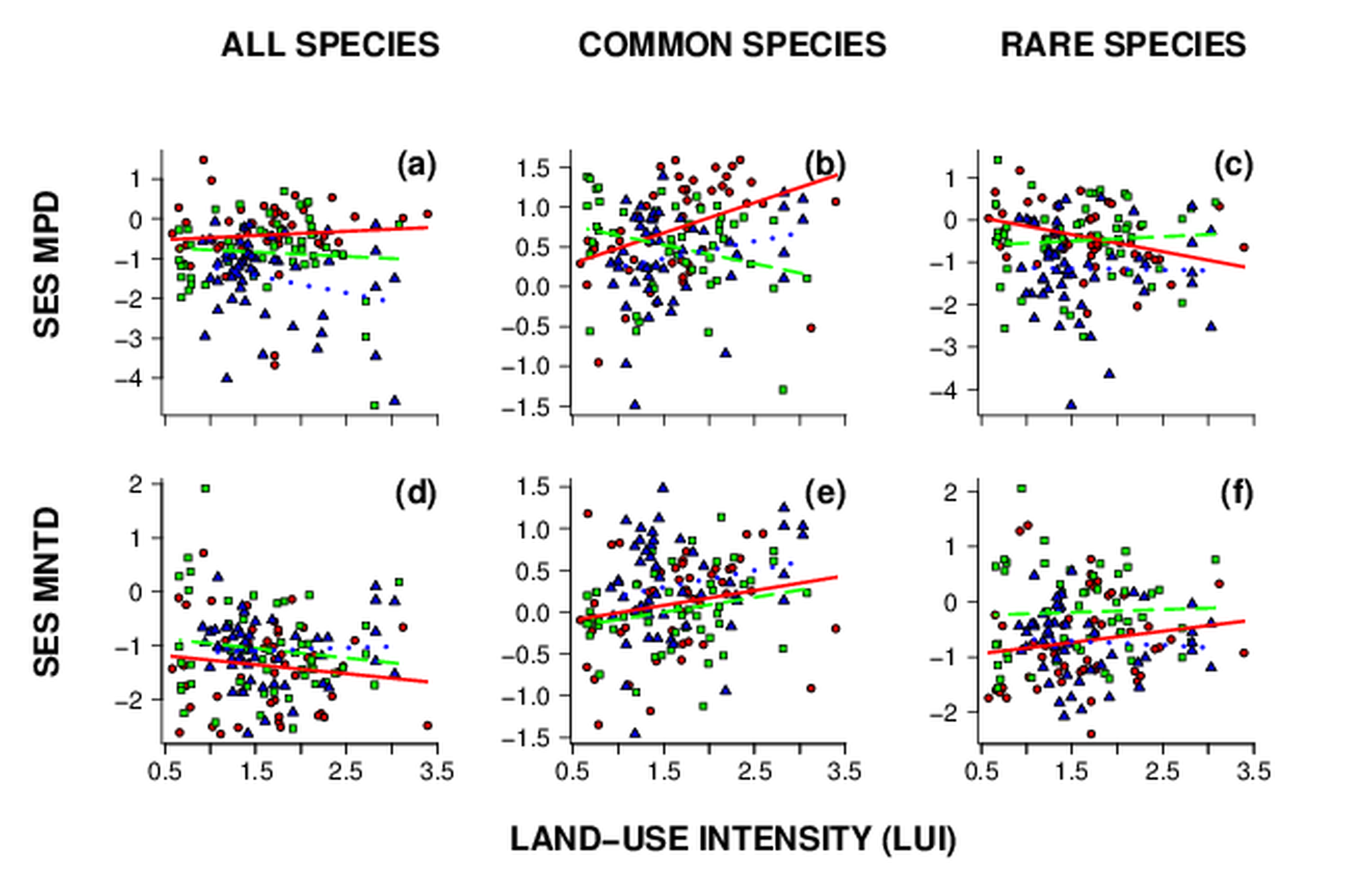

Supplement: Appendix S5 — Relationships between presence/absence based mean pairwise distance (effect size MPD), mean nearest taxon distance (effect size MNTD) and land-use intensity (LUI) in three regions in Germany. (TIF) [file pone.0103252.s005.tif]

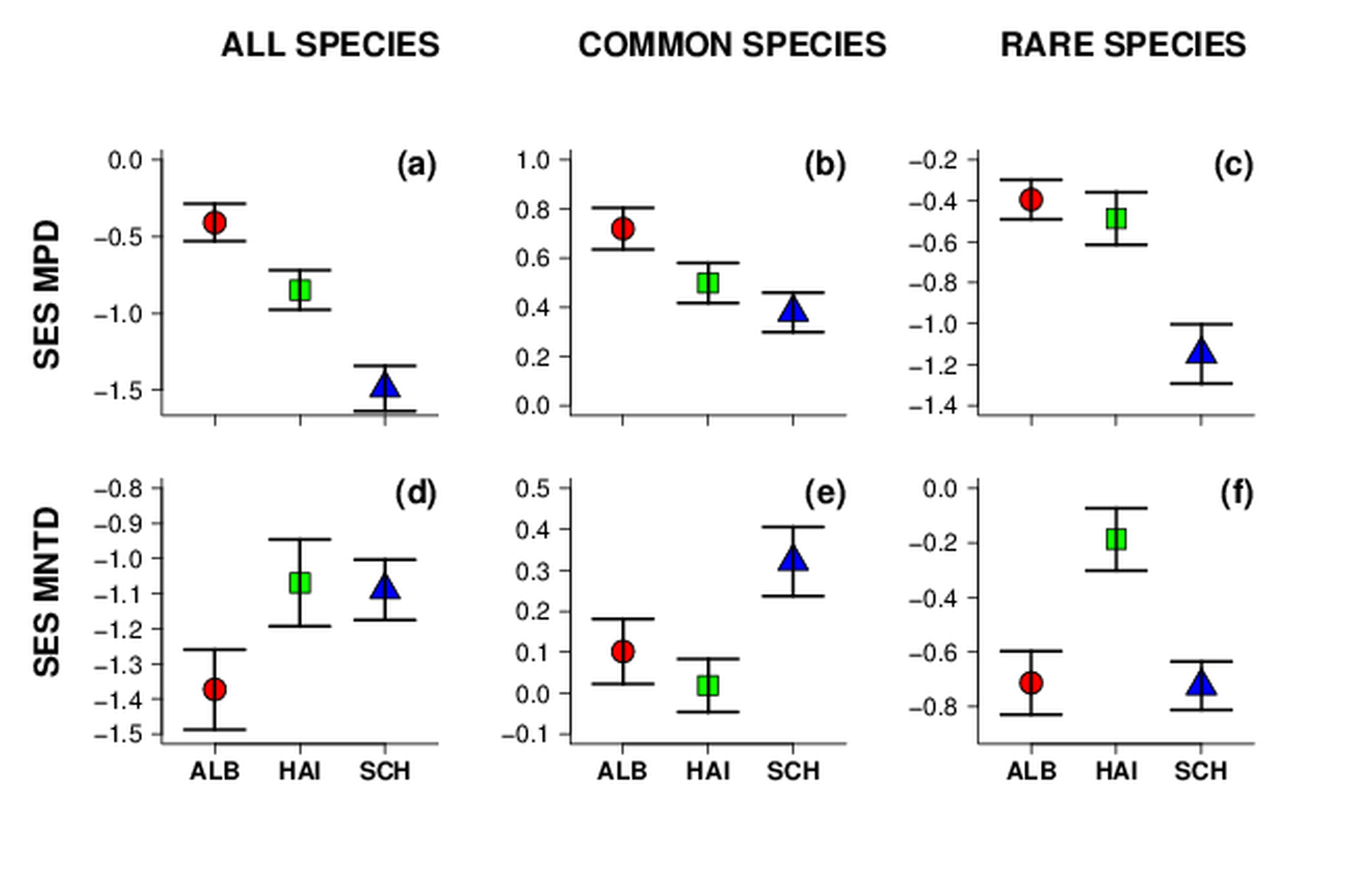

Supplement: Appendix S6 — Mean (±SE) values of presence/absence based MPD and MNTD effect sizes for total, common and rare species assemblages in three regions in Germany. (TIF) [file pone.0103252.s006.tif]
