# Supplementary material for: Does Land-Use Intensification Decrease Plant Phylogenetic Diversity in Local Grasslands?
Source: PLoS One. 2014 Jul 25;9(7):e103252. doi: 10.1371/journal.pone.0103252 (PMC4111588; doi:10.1371/journal.pone.0103252)
Supplement: Appendix S4 — Correlation coefficients and significance of the respective regression slopes from Fig. 1 and Appendix S3 and for all regions combined. (DOCX) [file pone.0103252.s004.docx]

**Table S4. Correlation coefficients of species richness, MPD and MNTD of all, common and rare species and LUI in the three regions (ALB: Schwäbische Alb, HAI: Hainich-Dün and SCH: Schorfheide-Chorin) and in all regions combined.**

| \| **SPECIES RICHNESS** \| \| \| \| \| \| \| \| --- \| --- \| --- \| --- \| --- \| --- \| --- \| \|  \| **ALL** \| \| **COMMON** \| \| **RARE** \| \| \| **ALL** \| 0.51 \| *** \| 0.06 \|  \| -0.54 \| *** \| \| **ALB** \| -0.68 \| *** \| 0.45 \| *** \| -0.68 \| *** \| \| **HAI** \| -0.74 \| *** \| -0.53 \| *** \| -0.72 \| *** \| \| **SCH** \| 0.02 \|  \| 0.03 \|  \| 0.01 \|  \| \|  \|  \|  \|  \|  \|  \|  \| \| **MPD** \| \| \| \| \| \| \| \|  \| **ALL** \| \| **COMMON** \| \| **RARE** \| \| \| **ALL** \| -0.15 \| . \| -0.009 \|  \| -0.15 \| . \| \| **ALB** \| -0.13 \|  \| 0.03 \|  \| -0.03 \|  \| \| **HAI** \| -0.07 \|  \| 0.004 \|  \| -0.07 \|  \| \| **SCH** \| -0.3 \| * \| -0.08 \|  \| -0.33 \| * \| \|  \|  \|  \|  \|  \|  \|  \| \| **MNTD** \| \| \| \| \| \| \| \|  \| **ALL** \| \| **COMMON** \| \| **RARE** \| \| \| **ALL** \| -0.2 \| * \| -0.08 \|  \| 0.09 \|  \| \| **ALB** \| -0.39 \| ** \| -0.12 \|  \| 0.15 \|  \| \| **HAI** \| -0.08 \|  \| -0.01 \|  \| 0.29 \| * \| \| **SCH** \| -0.12 \|  \| -0.15 \|  \| -0.26 \| . \| | | | |  |
| --- | --- | --- | --- | --- | --- | --- | --- | --- | --- | --- | --- | --- | --- | --- | --- | --- | --- | --- | --- | --- | --- | --- | --- | --- | --- | --- | --- | --- | --- | --- | --- | --- | --- | --- | --- | --- | --- | --- | --- | --- | --- | --- | --- | --- | --- | --- | --- | --- | --- | --- | --- | --- | --- | --- | --- | --- | --- | --- | --- | --- | --- | --- | --- | --- | --- | --- | --- | --- | --- | --- | --- | --- | --- | --- | --- | --- | --- | --- | --- | --- | --- | --- | --- | --- | --- | --- | --- | --- | --- | --- | --- | --- | --- | --- | --- | --- | --- | --- | --- | --- | --- | --- | --- | --- | --- | --- | --- | --- | --- | --- | --- | --- | --- | --- | --- | --- | --- | --- | --- | --- | --- | --- | --- | --- | --- | --- | --- | --- | --- | --- | --- | --- | --- | --- | --- | --- | --- | --- | --- | --- | --- | --- | --- | --- |
|  |  |  |  | |

*** = p<0.001; ** = p<0.01; * = p<0.05; . = p<0.1
